# Technology and provenience of the oldest pottery in the northern Pannonian Basin indicates its affiliation to hunter-gatherers

**DOI:** 10.1038/s41598-024-69208-7

**Published:** 2024-08-20

**Authors:** Jan Petřík, Karel Slavíček, Katarína Adameková, Victory A. J. Jaques, Martin Košťál, Peter Tóth, Libor Petr, Dalibor Všianský, Tomas Zikmund, Jozef Kaiser, Jozef Bátora, Penny Bickle

**Affiliations:** 1 Department of Geological Sciences, Faculty of Science, Masaryk University, Kotlářská 2, 611 37 Brno, Czechia; 2 Institute of Archaeology of the Czech Academy of Sciences, Brno, Čechyňská 363/19, 602 00 Brno, Czechia; 3 CEITEC ‑ Central European Institute of Technology, Brno University of Technology, Purkyňova 123, 612 00 Brno, Czechia; 4 Department of Archaeology and Museology, Faculty of Arts, Masaryk University, Arna Nováka 1, 602 00 Brno, Czechia; 5 Department of Botany and Zoology, Faculty of Science, Masaryk University, Kotlářská 2, 611 37 Brno, Czechia; 6 Institute of Archaeology, Slovak Academy of Sciences, Akademická 2, 949 21 Nitra, Slovakia; 7 Department of Archaeology, University of York, The King’s Manor, York, YO1 7EP UK

**Keywords:** Hunter-gatherers, Pottery technology, Provenience, Pottery firing, Organic temper, Microtomography, Plant sciences, Geology, Ceramics

## Abstract

Consensus holds that pottery technology came to Central Europe from the Northern Balkans with independent pottery traditions existing concurrently in Eastern Europe. An unusual grass-tempered pottery dating back to around 5800 cal BC found in lake sediments at Santovka, Slovakia, predated the earliest known Neolithic pottery in the region (~ 5500 cal BC), suggesting unexplored narratives of pottery introduction. Analyses of the pottery’s technology, origin, and grass temper shedding light on ceramic traditions' spread can unveil mobility patterns and community lifestyles. Our findings indicate a non-local provenance, low temperature firing, *Festuca* sp. grass temper and unique rectangular or cylindrical vessel shapes which align with Eastern European hunter-gatherer practices. Moreover, the pottery style and technology have no analogies in the contemporary Danubian pottery traditions and have more similarities to those of the Eastern traditions. The pottery's raw materials likely originated from distant areas, indicating extensive territorial access for its creators. Our findings imply late Mesolithic hunter-gatherers as the probable artisans and with implications for the site's significance in the late Mesolithic landscape.

## Introduction

**Figure 1:**
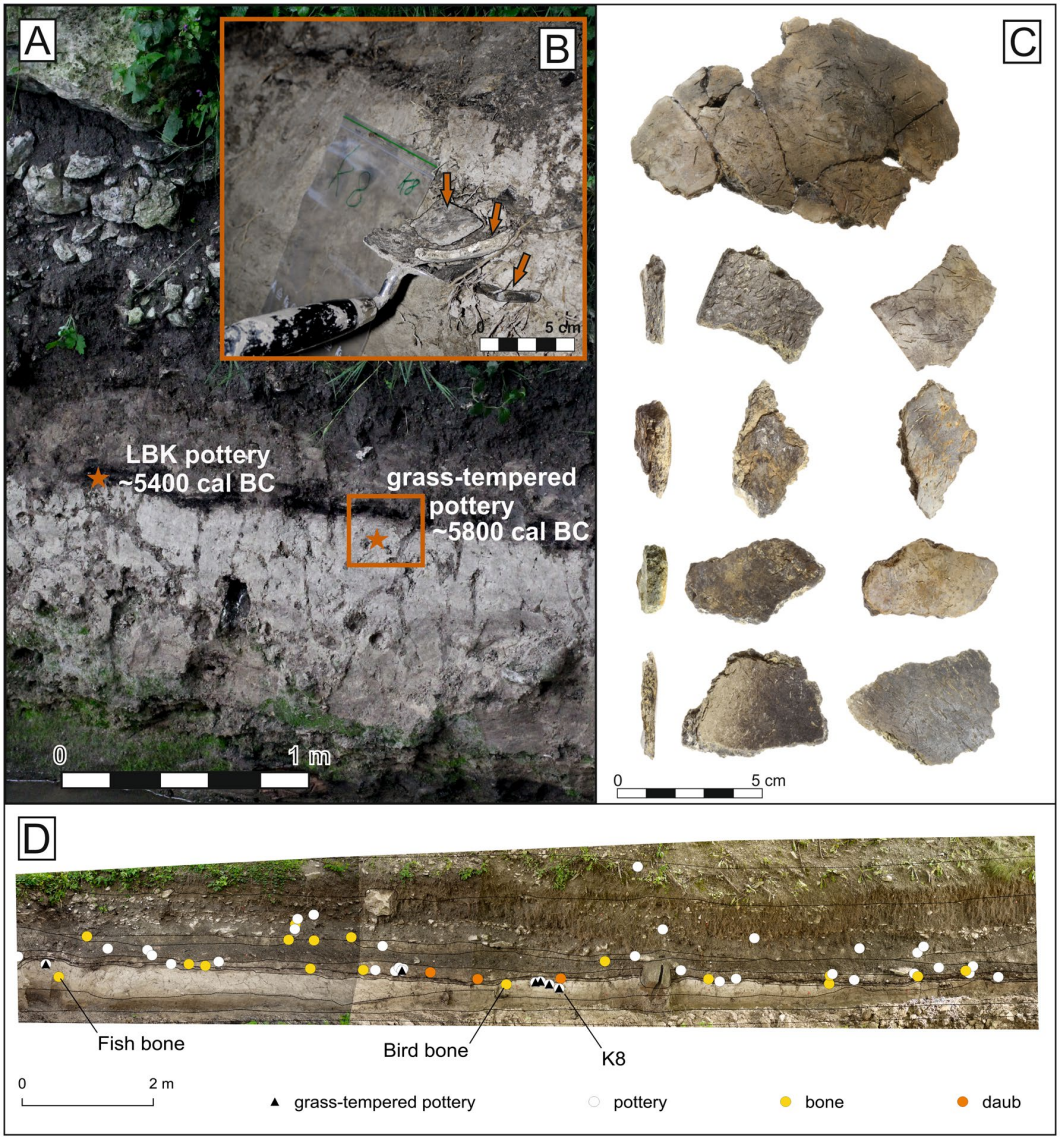
The sediment section at the Santovka site with the depiction of LBK pottery's position and, stratigraphically below, the grass-tempered pottery, along with the approximate results of radiocarbon dating (**A**). Detailed photograph showing the stratigraphic position of the grass-tempered pottery (**B**). Selection of grass-tempered pottery fragments (**C**). Uncovered and cleaned profile in the Búr stream cut (Santovka village) with marked positions of finds (animal bones, daub, and flint), indicating the position of grass-tempered pottery in the light lake sediments (**D**).

**Figure 2:**
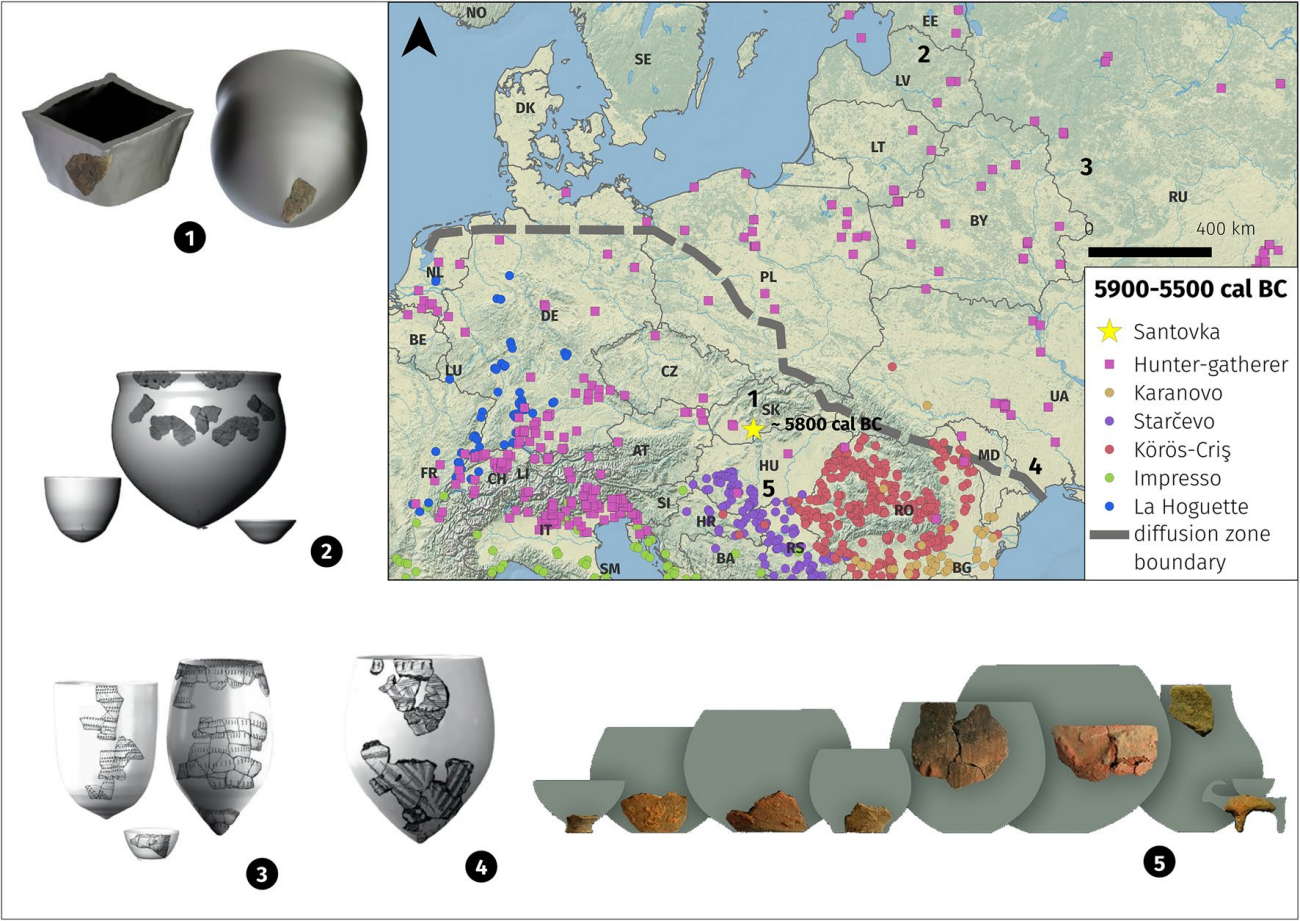
Cultural-historical description of the first half of the 6th millennium BC and major pottery styles in the region. Assumed shapes of grass-tempered pottery from Santovka made by 3D modelling (1). Hunter-gatherer pottery shapes from Eastern Baltic (2), Upper Dnieper (3) and Bug-Dniester (4) regions. Pottery shapes of Starčevo-Körös-Criş culture (5). Grey line marks the boundary between the diffusion zones of farming and hunter-gatherer pottery tradition^5–8^. Points indicate Neolithic sites, squares indicate hunter-gatherer sites, basemap is from Natural Earth.

An unusual grass-tempered pottery (Fig. 1) dated ca. 5800 cal BC^1^ was discovered recently in Santovka (Slovakia) situated at the transition between the Pannonian Basin and Western Carpathians (Fig. 2). The site itself is known for the occurrence of springs and related travertine mounds. Likely because of this, Santovka became a centre of human activities from the Paleolithic^2^, with increasing human activity during the Neolithic^3^ and the Bronze Age^4^. Pottery was found in the section of lake carbonate sediments (Fig. 1) excavated 2011–2014 close to one of the travertine accumulations. As pottery was stratigraphically sealed under the Neolithic layer bearing exclusively the Early Neolithic pottery. The sealing layer including pottery shards were radiocarbon dated after to the mid-6th millennium BC. In conclusion the grass tempered pottery precedes the onset of the oldest pottery known to date in Central Europe^1^. This new pottery type suggests that there are hitherto unrecognised gaps in our knowledge about the introduction of pottery into Central Europe.

Pottery vessels were invented in East Asia between 19.2 and 15.9 ka cal BC^9^ by hunters-gatherers, who dispersed the ceramic technology during the Pleistocene-Holocene transition^10^. Around 7000–6800 cal BC the pottery was introduced to the Near East, where it became an integral part of the Neolithic way of life^11–13^. The spread of ceramic technology among European communities occurred in two distinct diffusion zones during the 7th and 6th millennium cal BC^5^. The first zone belongs to Eastern Europe’s hunter-gatherers (i.e. the Eastern tradition). Recent studies suggest that the pottery arrived in the Dnieper and Bug catchments, East Baltic zone and northern Poland before ca. 5500 cal BC;^6,14^. In contrast, the second diffusion zone (i.e. the Danubian tradition) in South-eastern Europe is associated with farming communities in the Northern Balkans and Pannonian Basin (e.g. Starčevo, Körös, Criş and consequent LBK) and is tied to an Aegean–Anatolian origin^15^. The pottery appears in the south Balkans after 6400 cal BC^16^.

The Santovka site is situated at the margin of the above-mentioned zones, so the age of pottery alone cannot answer questions about its origin and who made it. As farming spread west with the Linearbandkeramik (LBK, ca. 5600–5000 cal BC) culture, researchers have identified distinct pottery styles that are unrelated to the LBK tradition. These wares, mostly tempered with shell or bone and featuring pointed bases wares, are classified as the “so-called hunter-gatherer” potteries of La Hoguette, Limburg, and Begleitkeramik, among others^17^. These fabrics are generally thought to represent indigenous hunter-gatherer groups that were present before and alongside migrating farmers. However, their precise role remains debated, and the classification of these groups as hunter-gatherer or farmer based on their pottery has been challenged^18^. Hence, the need to focus on the provenience and technology of this unusual pottery to fully situate it within the spread of different ceramic traditions.

The pottery of the Danubian tradition in the central and northern Balkans, encompassing the Great Hungarian Plain, features an abundant use of plant temper^19^. This organic material primarily consists of by-products from cereal production, especially chaff^20^. The eastern tradition, in contrast, does not rely on cereal remnants. Instead, it uses various plant residues and further includes tempering materials such as minerals and rocks (sand, crushed granites, flints), shells, and occasionally grog^14,21–25^. This pottery appears to have been used for cooking meat, especially fish^25^.

Understanding the technology and provenance of Santovka pottery can shed light on the origin of ceramic technology north of the Balkan farming frontiers, while technological choices can inform on mobility and the way of life of the communities who produce it. To address this issue, we had four specific aims: (1) examination of provenance and clay preparation with petrographic and mineralogical methods, (2) identification of temper and pot shaping technique with use of microtomography, (3) vessel shape reconstruction based on 3D modelling, and (4) reconstruction of firing technology with implementation of firing experiment.

## Material and methods

A total of 25 body sherds and one bottom point were found in Santovka, and the total number of refits is 6. Fragments are ca. 5–10 mm thick, fragile and apart for one all without significant morphological features (MNI ~ 2, MNA = 19). Their colour is grey and brown, with lighter shades on the outer surface and dark grey to black along the fractures. The ceramic paste contains a significant amount of organic inclusions visible by the naked eye. Sherd surfaces bear no signs of decoration. Fragments were often smoothed on the outside. The inner and outer surface is covered with unoriented thin linear pores after vegetal inclusions (Fig. 1). Given fragmentary character, rarity and homogeneity of material the sampling strategy was designed accordingly to preserve the shards to the maximum extent.

In this study, various methods were employed to analyze six ceramic fragments. Thin sections were prepared and analyzed micro-petrographically under a polarizing microscope, following established protocols for inclusion and void abundance as well as organic content estimation. Scanning electron microscopy (SEM) was used to capture detailed images. X-ray powder diffraction (XRD) identified bulk phase composition, while thermal analysis (DSC/TG) quantified remaining organic matter. Total organic carbon (TOC) analysis measured charred organic content. Additionally, micro-computed tomography (μCT) visualized organic matter and porosity. A firing experiment was conducted to simulate ancient firing techniques. Detailed descriptions of these methods are provided in the Supplementary material.

## Results

### Characterization of raw material

**Figure 3:**
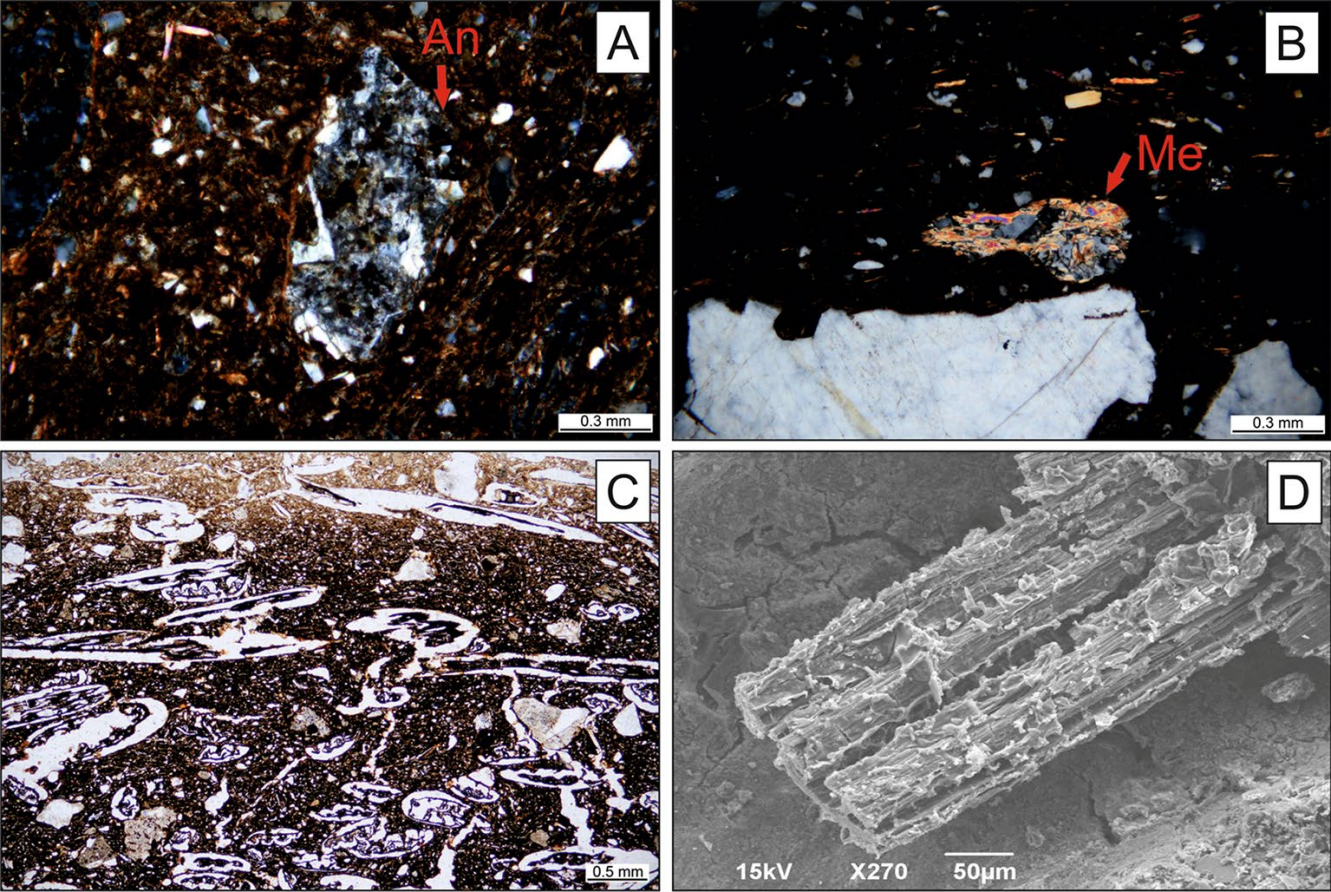
Microphotographs of grass tempered pottery. Rock inclusions of andesite (**A**) and metamorphic (**B**) rocks in cross-polarised light. Grass temper (*Festuca sp.)* of pottery in transmitted polarised light (**C**). SEM image of *Festuca *sp. (**D**).

All studied thin sections were similar in terms of petrographic characteristics, therefore no specific fabrics were determined. Ceramic matrix consists of clay with common, but not very abundant grains of silt. Aplastic inclusions are represented by common subangular to sub-rounded fragments of andesite (Fig. 3A) and quartz. Scarce rock fragments consisting of quartz, feldspars and micas were attributed to metamorphic rocks (Fig. 3B), possibly phyllites and gneisses. Plagioclase grains are more abundant than alkali feldspars. Mica flakes are occasional with more muscovite than biotite. Accessory minerals identified are rare to occasional amphiboles and scarce pyroxenes and epidote.

XRD analysis indicates the dominant mineral of is quartz (46.9–52.1 mass %) followed by mica minerals including illite (23.5–27.5%), and Na-Ca feldspars (21.5–22.5%), kaolinite (2.0–2.4%) and traces of K-feldspars (< 1%). The contents of the other accessory minerals was apparently below the limit of detection.

### Grass temper

**Figure 4:**
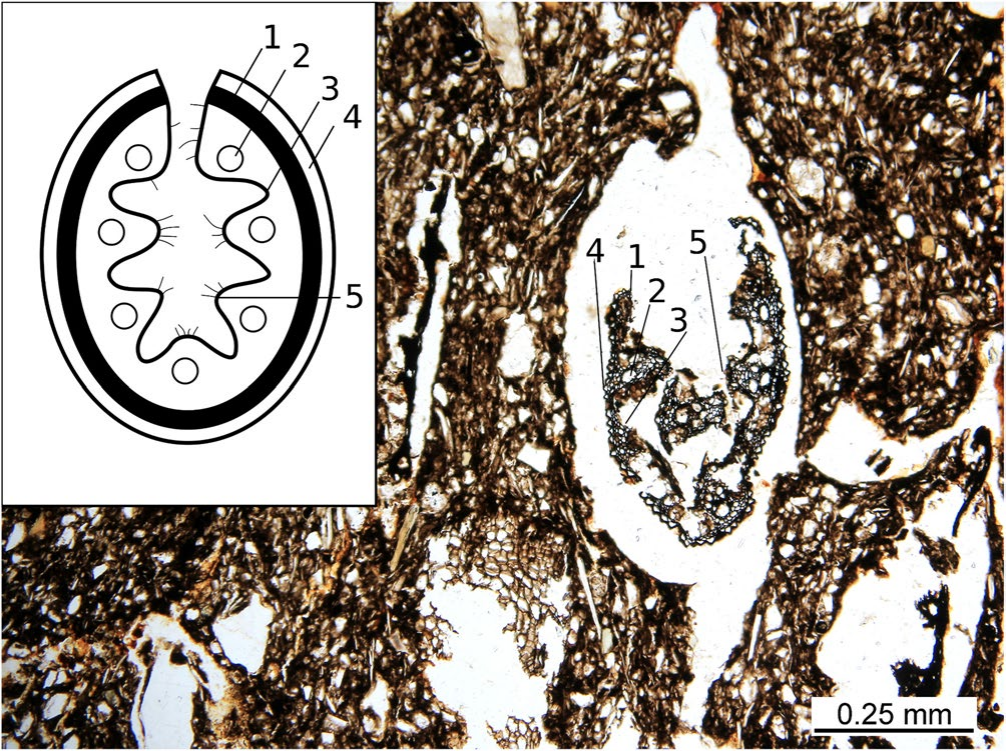
Festuca leaf cross-section scheme (modified after Martínez-Sagarra et al. 2017^26^) compared with microphotographs of preserved leaves in the pottery. The main observable features are sclerenchyma (1), vascular bundles (2) consisting of layers of bundle sheath cells, adaxial (3) and abaxial (4) epidermal cells and ribs with trichomes (5).

The ceramic paste was heavily tempered with grass (Fig. 3C). The organic content was surprisingly well-preserved in the dark, reduced parts of the sherd. The layer close to the surface, where enough oxygen was available, is oxidised, and the organic matter is completely burnt away. Numerous unburnt and charred parts were identified as fragments of stems and leaves of the narrow-leaved species of the Festuca genus, belonging to the Poaceae plant family commonly known as grasses (Figs. 3, 4). The preservation status of grass plant tissues allows for the identification of some morphological elements that make up the cross-section of a Festuca grass leaf. Usually, at least a part of the sclerenchyma is preserved, ribs are distinguishable, and sometimes even trichomes. Vascular bundles are sometimes visible, but are often collapsed. On the best-preserved sections, adaxial or abaxial epidermal cells can be discerned (Fig. 4). No chaff nor other features suggesting the presence of cereals were found.

**Figure 5:**
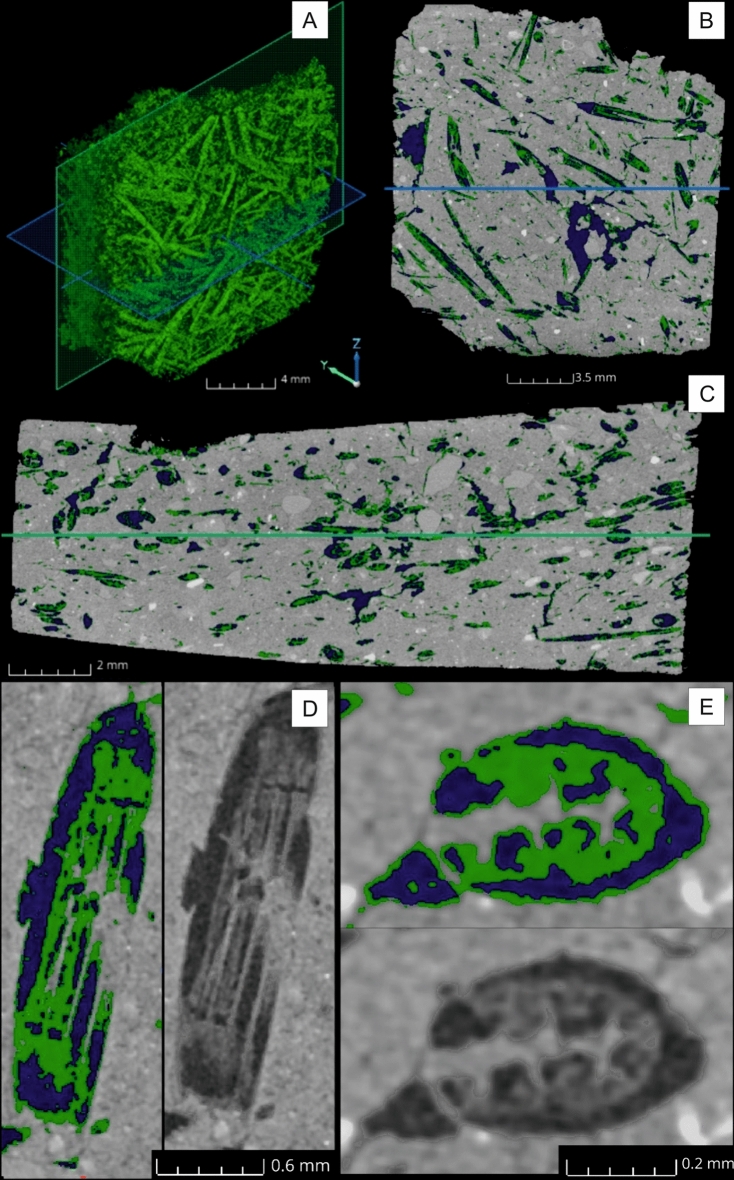
μCT (**A**) 3D visualisation of the organic matter in the ceramic sample K8 with XY oriented (green) and (**C**) XZ oriented slices positions (blue). The position of slice (**B**) and (**C**) is also shown in the other picture by a coloured line. Details of the same blade in a (**D1**) longitudinal orientation and (**E1**) perpendicular cross-section, where the organic matter is represented in green and air in dark blue. (**D2**) and (**E2**) show the same areas without segmentation, and with measured grey values (8-bit).

The orientation of grass temper visualised by μCT indicates sub parallel orientation in cross-section view and unparallel orientation in side view orientation Fig. 5A–C). From the CT dataset fibre orientation module, approximately 65% of the Festuca stems and leaves have a similar preferential orientation usually oriented parallel to the pot surface. The orientation of grass stems and leaves suggest that not coiling, but rather moulding or slab building are the case of Santovka pottery. Depending on the orientation of stems and leaves (Fig. 5D), the charred organic matter is present in oval and V-like feature forms respectively (Fig. 5E). When the orientation is parallel to the thin section plane, the grass forms an elongated feature. In certain samples, most of the organic material is oriented, and the used grass was probably mostly green, as pores are much larger than the preserved fragments after firing. Total porosity estimate is 20–30%.

Total organic carbon content after firing was 20.2 mg/g (2.02 mass %). The thermogravimetric (TG) experiment showed the total content of organic matter ca 3.3 mass %. The comparison of this value and TOC content corresponds to not completely carbonized plant mass. According to the shape of voids we assume that the former grass temper volume was much higher than the result of TOC. The amount of residual organic matter in the sherds from Santovka was quantified around 5% with a high standard deviation at ± 2.3%, while the total porosity (organic matter + void) was 14% (± 2.8%) from the CT analyses. The matrix is homogeneous in some samples and heterogeneous in others, where clay parts are more prevalent in certain areas and grass more in others. We can observe the complexity of the segmentation due to a relative low contrast between air and organic materials and sensible noise. We also observe a clear shrinkage between the original pore shape and the residual organic matter.

### Pottery shape and forming techniques

**Figure 6:**
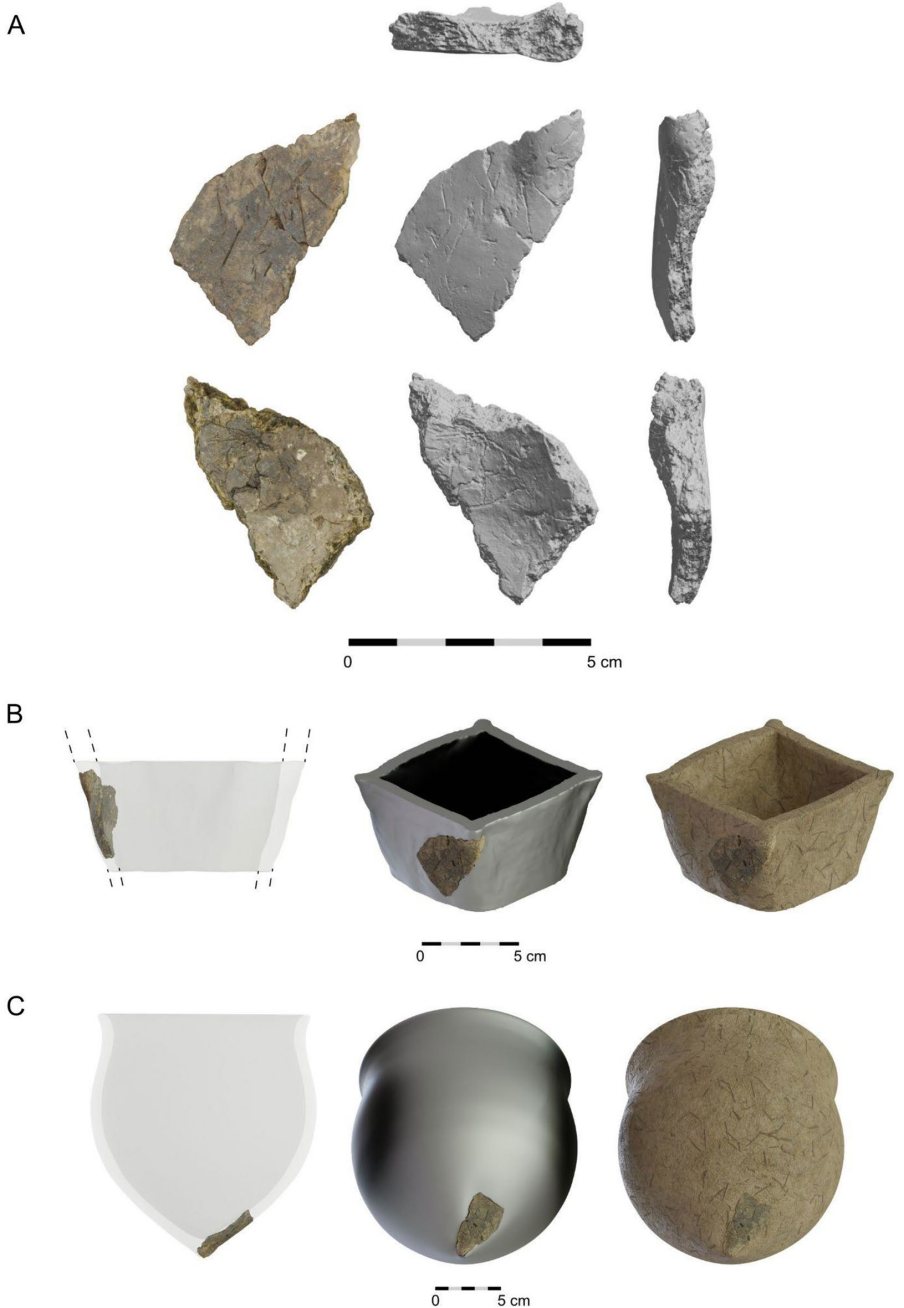
3D visualisation of (**A**) a peculiarly curved fragment found in Santovka and hypothetical variations of shape reconstruction based on pottery from hunter-gatherer contexts of (**B**) the Far East^9^ and (**C**) Eastern Europe^14^.

Only one bent sherd bore significant morphological features (Fig. 6A), which would allow for pot shapes to be determined. It was not possible to use strict metric methods to create a virtual reconstruction of the possible shape of the vessel from which the fragment originated, mainly due to the fragments small size. However, the shape specificity allowed the fragment to be placed in two morphologically specific vessels. The two interpretational models were based on thorough investigation of a 3D model of Santovka sherd: one possible shape is rectangular (Fig. 6B), the other cylindrical with a pointed base (Fig. 6C). The pointed base vessels are quite frequent in the Late Mesolithic contexts (e.g. Bug-Dniester and Dniepr-Dvina regions).

### Reconstruction of firing technology and experimental firing

**Figure 7:**
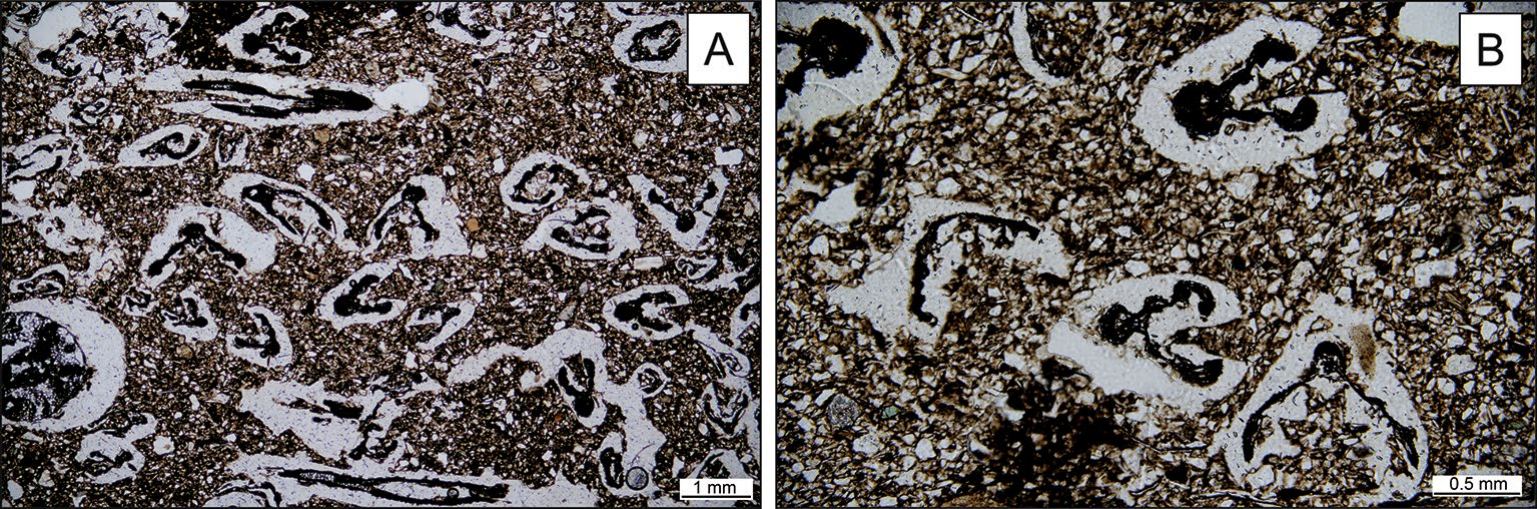
Microphotographs of cross and parallel sections of unoriented stems and leaves in a silty ceramic matrix of pottery from firing experiment (**A**). The detail on the Festuca sp. Temper from experiment (**B**).

The entire experimental firing process lasted until all the firewood burnt out naturally, which took four and a half hours. The temperature in the pit rapidly rose to the maximum measured temperature (just over 800 °C). This maximum temperature was reached approximately after one hour. Afterwards, it started to slowly and regularly decreased by about 200 °C per hour. The average maximum temperature measured on the surface of the vessels was 600 °C. The pottery sherds appear dark brown to black in the core which are indicators of a reducing environment. Ochre to light brown layer of oxidised matrix appears close to the surface of the pot. Pots of comparable appearance resulted from the experiment. The analysis of the thin sections of experimentally fired pottery show that the Festuca leaves and stems were mostly burnt out in the surface layer of the majority of experimental vessels, for they were oxidised on the surfaces (Fig. 7A). In contrast the cores were reduced allowing some amount of organic matter to be preserved. The leaves of Festuca grass in the ceramic core are carbonised, and individual morphological features are not easily distinguishable. The sclerenchyma is compacted and the adaxial or abaxial epidermal cells are indistinguishable. This is similarly the case for the vascular bundles, which can only be identified in some instances. Ribs are occasionally visible, and in rare cases, trichomes have been preserved (Fig. 7B).

## Discussion

**Figure 8:**
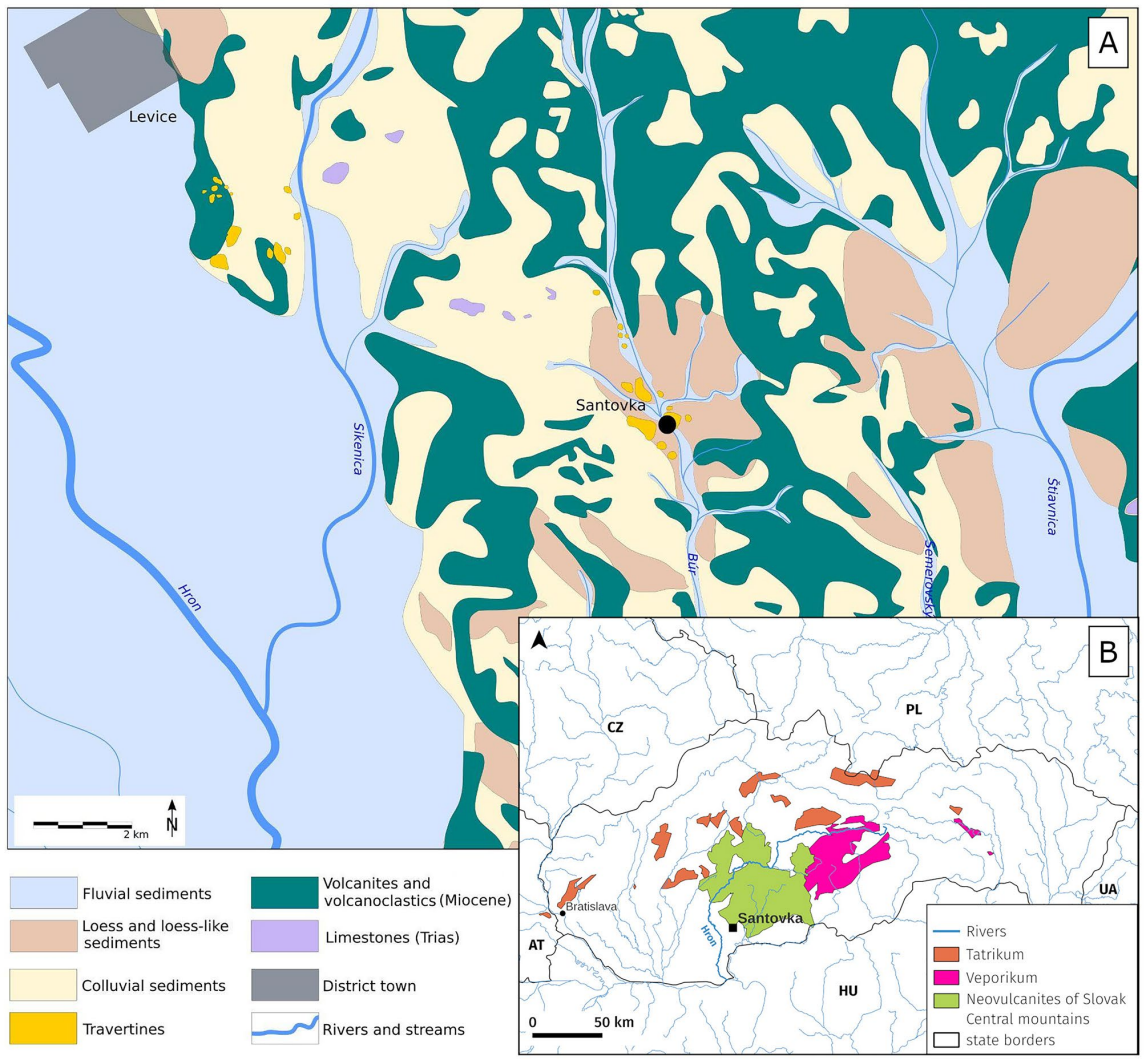
A geological map of the Santovka area^35^ and floodplains of Hron, Sikenica and Štiavnica rivers (**A**). Map of Slovakia with Hron river and source geological units of Neovulcanites and crystalline rocks (**B**).

The presence of volcanic rocks, particularly andesite, within the analysed material suggests a connection to the Slovak Central Mountains, which extend from the north of the site. The surrounding bedrock (Fig. 8A) is formed by volcanites, volcaniclastics, dolomites and limestones^27^. Concurrently, the inclusion of metamorphic rock fragments, indicates that the inclusions are also sourced from the mountain ranges which belong to so-called crystalline massifs of tectonic units, such as the Tatrikum or Veporikum (Fig. 8B). Bearing this in mind with addition to their subangular to sub-rounded shape, the raw material used to produce the pottery was likely gathered from the catchment areas of a river, flowing from and around both volcanic mountain range and crystallines massifs of Inner Western Carpathians^28^. The nearest floodplain matching the mentioned prerequisites could belong to the Hron river flowing ca. 10 km to the west from the studied site of Santovka (Fig. 8A). A similar composition of inclusions was recorded in prehistoric pottery produced in the same river catchment, at the site Rybník located ca. 20 km up the stream^29^. Our results indicate that the raw material originates more than 10 km away from the site, what may suggest that the Santovka pottery was not produced at the place of find^30^, demonstrating the significant range of territory in which its producers moved or had access. It contrasts with the typical provenance of the pottery of the first farmers which were usually made from more local clay sources^20,31–34^.

The prevalent grass temper of Santovka pottery are leaves and stems from the narrow-leaved Festuca genus. Organic-tempered vessels appear from the onset of the Neolithic period in both the Middle East and the southern Balkans. There is evidence of grass glume phytoliths, sometimes identified as cereals^36^. North of these regions, organic-tempered pottery almost became synonymous with pottery by the late 7th and 6th millennium BC. Alongside the regional variability observed in the LBK, such as the use of moss in Western Europe^37^, manure^38^, or shells^39^, there are notable temporal and spatial similarities with the use of local fine-grained clays combined with by-products from cereal productions^19,20, 40, 41^. Increasing archaeological evidence suggests that pottery is not uncommon within the environment of hunter-gatherers^25^, yet the specific technological choices of hunter-gatherers' pottery in Eastern Europe are very diverse. Their tempering practices differ regionally; pottery in Pomerania (Poland) included coarse mineral temper of crushed granite^22^, in northern Poland with sand and plants^14^, in the Baltic it was quartzite, sandstone, mineral inclusions, flint, mollusks, less frequently grog (crushed pottery), vegetal temper^14,23^ and shells^25^, mollusks were added to pottery in eastern Ukraine^42^, shells, organic temper or grog in Eastern Europe^21^, bone, shell, minerals, and organics in “so-called hunter-gatherer” potteries from western Europe^17^, or grass in Far East^9^. This, however, does not include cereal remains common in the pottery of agricultural Neolithic cultures^21,25^.

Due to the fragment's small size, a virtual reconstruction of the vessel's original shape was challenging, however we suggest two possibilities. The box-like shape was identified in discoveries from e.g. Russian Far East^9^, based on net-like imprints on the external surface. These vessels were formed using baskets or cord bags, which served as molds. The resulting vessels were open-mouthed, straight-walled, and occasionally flat-bottomed (Fig. 6B). Finds from Sakhalin Island (7000–5500 BC) indicate the use of rectangular box-shaped containers. The cylindrical shape with a pointed base, which appears (presumably also in a squared variant) within the Bug-Dniester and Dniepr-Dvina regions during the 7th–6th millennium BC are analogues from a closer region^14,21^.

The good preservation state of grass temper in Santovka pottery could be associated with a very low firing temperature (~ 600 °C) and a short soaking time. Assumption of such a low firing temperature is also supported by: (1) the comparison of total organic carbon (2.02 mass %) and organic matter (3.3 mass %) contents, which correspond to not completely carbonized plant mass; and (2) the presence of kaolinite in the ceramics material. The undecomposed kaolinite indicates even lower temperatures (< 550 °C)^43^. Pottery of the Neolithic cultures with Danubian origin was fired at a temperature that did not exceed 750–800 °C^20,31–34^. In comparison, traits of early Eastern tradition pots are found in limited quantities, as highly fragmented sherds from low firing temperatures ranging from 450 to 600 °C, suggesting that vessels were fired on open hearths^9,22^, which corresponds to our observations of Santovka’s pottery. The quantity of preserved organics and remaining voids suggests a high amount of grass temper (~ 14%), which also indicates functional differences. Using a larger amount of grass temper makes the pottery lighter, thereby increasing the portability of the vessels.

Low density of late Mesolithic settlements in areas of Transdanubia, Lower Austria and SW Slovakia^7,44–46^ supports an observation, that these loess areas settled by the incoming LBK were marginal to already scarce Mesolithic population, located in higher altitudes, near active river channels for mollusk gathering^47,48^. In the north of the Danube, the grass-tempered pottery of Santovka (ca. 5800 cal BC) predates the presence of the earliest known Neolithic contexts with pottery finds by approximately 200 years^1^. Moreover, contextual data (freshwater lake site, high fragmentation of pottery) and the technology in terms of used grass temper, low firing temperature and reconstructed shapes, similar to those of the Eastern ceramic tradition, support the idea that pottery was produced by Late Mesolithic hunter-gatherers.

**Figure 9:**
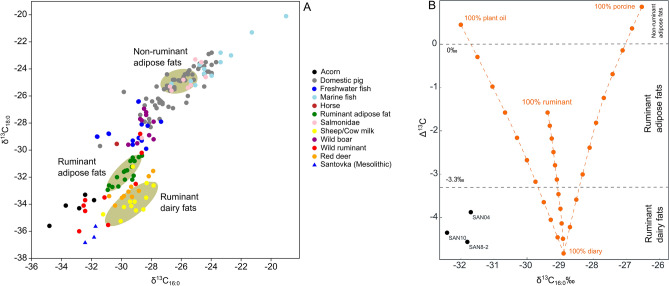
(**A**) The plot presents the δ^13^C values of C_16:0_ and C_18:0_ n-alkanoic acids extracted from Mesolithic pottery at the Santovka site^1^. The beige ellipses represent different food commodity groups, which are determined based on the δ^13^C values of reference animal fats from domesticated animals known in British prehistory^57^. The connecting lines between the ellipses illustrate theoretical mixing curves, demonstrating the potential effects of vessel re-use and the processing of mixed food commodities. The reference values used in this study are derived from various sources, including Copley et al.^57^, Craig et al.^58^, personal communications with Craig and Isaksson, as well as studies by Dudd et al.^59^, Evans et al.^60^, and Lucquin et al.^61^. (**B**) The difference in δ^13^C isotope values (Δ^13^C = δ^13^C_18:0 _– δ^13^C_16:0_) for C_16:0_ and C_18:0_ n-alkanoic acids extracted from Mesolithic pottery (black) at the Santovka site. The orange lines represent theoretical mixing curves of δ^13^C_16:0_ and Δ^13^C values, which are calculated by mixing modern dairy, plant oils, porcine fat, and ruminant carcass fat. The filled circles on these curves indicate 10% increments between mean values^60^. The Δ^13^C values can be used to identify the source of the fats: values below − 3.3‰ are typically associated with ruminant dairy fats, while values between − 3.3 and 0‰ correspond to ruminant adipose fats. Δ^13^C values above 0‰ are generally linked to non-ruminant adipose fats.

The conclusion is supported as well by results of lipid analysis pointing to cooking to a mixture of wild ruminants and plants (Fig. 9). Eurasian hunter-gatherers and the first farmers used pottery differently; aquatic products are largely absent in the pottery produced by the first farming communities, who instead preferred ruminants and dairy sources^49–51^. Researchers argue that the appearance of pottery in hunters-gatherers was tied with the seasonal intensification of aquatic resource exploitation in the Late Mesolithic period, linked with broadening subsistence systems, increased sedentism and population growth^10,52–54^. The evidence of terrestrial resource exploitation at Santovka highlights the increasing diversification of subsistence strategies during the late Mesolithic period (Fig. 9). The diverse and multifaceted nature of hunter-gatherer subsistence strategies precludes the notion of a uniform dietary pattern across pre-Neolithic Europe, just as the variability in agricultural practices renders a monolithic concept of a single Neolithic diet untenable^55,56^. While our isotopic analysis did not capture evidence of fish consumption, previous paleoecological research indicated the presence of numerous fish bones in the holocene sediments at the site^3^, suggesting the availability of aquatic food sources.

Some researchers propose that hunter-gatherer pottery was not widely used for everyday activities, but instead served as prestigious item linked to power and status^11^ or held sacred significance^21^. The riverine location of Santovka, situated near mineral springs and a lake/natural pond, further suggests that this site held a special importance or served a unique purpose within the late Mesolithic landscape. The pond contained remains of large birds and fish, as indicated by the findings (Fig. 1).

Based on Jordan et al.’s spatio-temporal model^5^, Santovka is close to the boundary dividing dispersal zones associated with farming (south of that boundary) and with hunter-gatherer (north of the boundary) traditions, and the timing also fits with this model. However, it does not entirely fit with a more recent model^6^, which found that pottery spread more quickly among Eastern European hunter-gatherers than previously believed, as Santovka belongs to the area where, at least according to the model, pottery was introduced last. The pottery dates from Santovka suggests an even more rapid and non-linear dispersal as it is contemporary with Eastern Europe hunter-gatherer sites like Melnychna Krucha (north-western Pontic region;^62,63^. This model also chimes with Kirschneck’s^17^ analysis of non-LBK ceramic traditions in the western regions, which suggests that technical decisions were made independent from both the LBK and Mediterranean Cardial ceramic technology traditions (the latter often cited as having influenced decorative methods^64^), leading him to suggest these diverse groups of ceramics were made by hunter-gatherers. Considering both the geographical and temporal place of Santovka^1^, drawing direct parallels or hunting for shared origins with the potteries associated with the western distribution of the LBK is inappropriate. However, their presence in the archaeological record does help to establish that ceramics independent of the main migration of farming communities were made, likely by a diverse group of communities, further supporting the proposed hypothesis that the ceramics at Santovka are an independent hunter-gatherer made technology.

## Conclusion

By focusing on the provenience and technology of this distinctive pottery, we are better positioned to understand the origin of pottery north of the Balkan farming frontiers and identify the specific human communities involved. The technology of Santovka pottery, including the use of grass temper, low firing temperature (below 600 °C), and estimated shape interpretations, indicates a stronger affiliation with mid-Holocene Eastern European hunter-gatherers. Moreover, the non-local origin of Santovka pottery suggests an affiliation with mobile groups of people, contrasting with the typical provenance of the first farmers' pottery of the Danubian tradition. Instead, parallels emerge with the pottery technologies of Eastern Europe, where the use of organic materials and similar shapes began before the spread of farming. Located at the intersection of two major diffusion zones from the 7th and 6th millennium cal BC, the Santovka site challenges our pre-existing assumptions about pottery introduction into Central Europe. Future extensive and in-depth studies are essential to validate the model proposed in this article, given the limited size of the Santovka corpus and the sparse data on local Late Mesolithic groups.

## Supplementary Information

Supplementary Information.

## Data Availability

The datasets used and/or analysed during the current study available from the corresponding author on reasonable request.

## References

[1] Tóth, P. *et al.* Radiocarbon dating of grass-tempered ceramic reveals the earliest pottery from Slovakia predates the arrival of farming. *Radiocarbon* **65**(3), 733–753. 10.1017/RDC.2023.39 (2023).

[2] Bárta, J. Nové poznatky o paleolitickom osídlení južného Slovenska. *Anthropos* **14**, 167–171 (1961).

[3] Šolcová, A. *et al.* Early and middle Holocene ecosystem changes at the Western Carpathian/Pannonian border driven by climate and Neolithic impact. *Boreas* **47**(3), 897–909. 10.1111/bor.12309 (2018).

[4] Bátora, J., Tóth, P. & Bača, M. Výskumy opevnených sídlisk zo staršej doby bronzovej vo východnej časti Podunajskej nížiny. In *Keď Bronz Vystriedal Meď**, **Archeologický ústav SAV Nitra* (eds. Bátora, J. & Tóth, P.) 139–154 (Katedra archeológie FiF UK Bratislava, Nitra, 2015).

[5] Jordan, P. *et al.* Modelling the diffusion of pottery technologies across Afro-Eurasia: Emerging insights and future research. *Antiquity* **90**(351), 590–603. 10.15184/aqy.2016.68 (2016).

[6] Dolbunova, E. *et al.* The transmission of pottery technology among prehistoric European hunter-gatherers. *Nat. Hum. Behav.* **7**, 171–183 (2023). 36550220 10.1038/s41562-022-01491-8 PMC9957732

[7] Stadler, P. & Kotova, N. S. (eds.) Early Neolithic settlement Brunn am Gebirge, Wolfholz, in Lower Austria. Volume 1. Langenweißbach Wien: Beier & Beran (Beiträge zur Ur- und Frühgeschichte Mitteleuropas, 2019).

[8] Jakucs, J. *et al.* Between the Vinča and Linearbandkeramik Worlds: The diversity of practices and identities in the 54th–53rd centuries cal BC in Southwest Hungary and Beyond. *J. World Prehistory* **29**, 267–336 (2016). 10.1007/s10963-016-9096-x PMC5040754 27746586

[9] Zhushchikhovskaya, I. S. Pottery making in prehistoric cultures of the Russian far east. In *Ceramics Before Farming* (eds Jordan, P. & Zvelebil, M.) 121–148 (Left Coast Press, 2009).

[10] Jordan, P. D. & Zvelebil, M. Ex Oriente Lux: The prehistory of hunter gatherer ceramic dispersals. In *Ceramics Before Farming: The Dispersal of Pottery Among Prehistoric Eurasian Hunter-Gatherers* (eds Jordan, P. D. & Zvelebil, M.) 33–89 (Routledge, 2009).

[11] Budja, M. Pots and Potters in the Mesolithic–Neolithic Transition in South-East Europe. In *The Oxford Handbook of Neolithic Europe *(eds. Fowler, C., Harding, J. & Hofmann, D.), 535–553 (Oxford University Press, 2015). [Accessed 2021 Aug 16]. 10.1093/oxfordhb/9780199545841.001.0001/oxfordhb-9780199545841-e-028.

[12] Nieuwenhuyse, O. P., Akkermans, P. M. M. G. & van der Plicht, J. Not so coarse, nor always plain: The earliest pottery of Syria. *Antiquity* **84**(323), 71–85. 10.1017/S0003598X00099774 (2010).

[13] Özdoğan, M. Earliest use of pottery in Anatolia. In *Early Farmers, Late Foragers, and Ceramic Traditions: On the Beginning of Pottery in the Near East and Europe* (ed. Gheorghiu, D.) 22–43 (Cambridge Scholars Publishing Lt, 2009).

[14] Andreev, K. M. & Vybornov, A. A. Ceramic traditions in the forest-steppe zone of Eastern Europe. *Open Archaeol.* **7**(1), 705–717 (2021).

[15] Lipson, M. *et al.* Parallel palaeogenomic transects reveal complex genetic history of early European farmers. *Nature* **551**, 368–372 (2017). 29144465 10.1038/nature24476 PMC5973800

[16] Perlès, C. *The early Neolithic in Greece: The First Farming Communities in Europe* (Cambridge University Press, 2001).

[17] Kirschhneck, E. The phenomena La Hoguette and Limburg: Technological aspects. *Open Archaeol.* **7**(1), 1295–1344. 10.1515/opar-2020-0195 (2021).

[18] Hofmann, D. The changing role of “hunter-gatherer ceramics” in an LBK context. In *Something Out of the Ordinary? Interpreting Diversity in the Early Neolithic Linearbandkeramik and Beyond* (eds Amkreutz, L. *et al.*) 191–224 (Cambridge Scholars Publishing, 2016).

[19] Spataro, M. A comparison of chemical and petrographic analyses of Neolithic pottery from South-eastern Europe. *J. Archaeol. Sci.* **38**, 255–269 (2011).

[20] Kreiter, A., Pető, Á. & Pánczél, P. Materialising tradition: Ceramic production in Early Neolithic Hungary. In *The Early Neolithic in the Danube-Tisza Interfluve* (ed. Bánffy, E.) 127–140 (Archaeopress, 2013).

[21] Mazurkevich, A. & Dolbunova, E. The oldest pottery in hunter-gatherer communities and models of Neolithisation of Eastern Europe. *Doc. Praehist.* **42**, 13–66 (2015).

[22] Czekaj-Zastawny, A., Kabaciński, J., Terberger, T. & Ilkiewicz, J. Relations of Mesolithic hunter-gatherers of Pomerania (Poland) with Neolithic cultures of central Europe. *J. Field Archaeol.* **38**(3), 195–209 (2013).

[23] Papakosta, V. Early Pottery Use Among Hunter-Gatherers Around the Baltic Sea. Doctoral thesis. Stockholm University, Stockholm (2020).

[24] Kotova, N., Demchenko, O. & Kiosak, D. Innovations of the beginning of the sixth millennium BC in the Northern Pontic steppe. *Open Archaeol.* **7**(1), 1529–1549. 10.1515/opar-2020-0185 (2021).

[25] Spataro, M., Oras, E., Lucquin, A. & Bērziņš, V. Hunter-fisher-gatherer pottery production and use at the Neolithic shell-midden of Riņņukalns, Latvia. *Antiquity* **95**(384), 1446–1463. 10.15184/aqy.2021.127 (2021).

[26] Martínez Sagarra, G. & Abad, P. & Alcaraz, J. A. Study of the leaf anatomy in cross-section in the Iberian species of Festuca L. (Poaceae) and its systematic significance. *PhytoKeys*. **83**, 43–74. 10.3897/phytokeys.83.13746 (2017). 10.3897/phytokeys.83.13746 PMC5624202 29033649

[27] Hók, J., Šujan, M., Sýkora, M. & Šipka, F. Geológia a tektonika levicko-stantovskej elevácie (juhozápadný okraj štiavnického stratovulkánu). *Geologické práce, Správy* **135**, 47–50 (2020).

[28] Lexa, *et al*. Geological Map of the Western Carpathians and Adjacent Areas (2000).

[29] Petřík, J. Petroarchaeological Research of Ceramic Production in the Area of Western Carpathians at the End of the Early Bronze Age. Dissertation thesis (Masaryk University Brno, 2017).

[30] Arnold, D. E. *Ceramic Theory and Cultural Process* (Cambridge University Press, 1985).

[31] Zsók, I., Szakmány, G., Kreiter, A., & Marton, T. A balatonszárszói újkőkori kerámia leletegyütes archeometriai viszgálata. In *Környezet – Ember – Kultúra. A Természettudományok És a Régészet Párbeszéde* 411–422 (eds. Kreiter, A., Pető, Á. & Tugya, B.). (Magyar Nemzeti Múzeum Nemzeti Örökségvédelmi Központ, 2012).

[32] Spataro, M. Continuity and change in pottery manufacture between the early and middle Neolithic of Romania. *Archaeol. Anthropol. Sci.* **6**, 175–197 (2014).

[33] Bente, K., Durini, S., Küsel, S., Kunert, I., Keilholz, S. & Hölzig, H. Firing conditions of LBK and SBK pottery from Eythra (Germany) by means of high-temperature analytics. In *Archäometrie Und Denkmalpflege 2019. Jahrestagung an Der Akademie Der Bildenden Künste Wien* (eds. Herm, C., Merkel, S., Schreiner, M. & Wiesinger, R.). Institut Für Naturwissenschaften Und Technologie in Der Kunst, 11.-14. September 2019: 167–170 (2019).

[34] Sauer, R. Petrographical and mineralogical analyses of pottery and clay raw materials from Brunn am Gebirge, Wolfholz. In *Early Neolithic settlement Brunn am Gebirge**, **Wolfholz, in Lower Austria*, Vol. 1 (eds. Stadler, P. & Kotova, N. S.) 475–512 (Beiträge zur Ur- und Frühgeschichte Mitteleuropas, 2019).

[35] Maglay, J. *et al.**Geologická mapa kvartéru Slovenska 1:500 000*. ŠGÚDŠ (2009).

[36] Papadakou, T., Kotsakis, K. & Urem-Kotsou, D. Distribution of organic-tempered pottery in Southeast Europe and the Near East: A complex picture. The case of Northern Greece. *Open Archaeol.* **7**(1), 1425–1443. 10.1515/opar-2020-0197 (2021).

[37] Teetaert, D., Boudin, M., Goemaere, E. & Crombé, P. Reliability of 14C dates of moss temper preserved In Neolithic pottery from the Scheldt River Valley (Belgium). *Radiocarbon* **62**(6), 1667–1678. 10.1017/RDC.2019.148 (2020).

[38] Neumannová, K. *et al.* Variability in coiling technique in LBK pottery inferred by experiments and pore structure micro-tomography analysis. *Archeol. Rozhl.* **69**(2), 172–186. 10.35686/AR.2017.11 (2017).

[39] Gomart, L., Constantin, C. & Burnez-Lanotte, L. Ceramic production and village communities during the Early Neolithic in north-eastern France and Belgium. Issues regarding tempers and pot-forming processes. In *Matières à Penser: Raw materials acquisition and processing in Early Neolithic pottery productions. Proceedings of the Workshop of Namur (Belgium), 29 and 30 May 2015 (Séances de la Société préhistorique française, 11)* (ed. Burnez-Lanotte, L.) 133–156 (Société préhistorique française, 2017).

[40] Gomart, L., Anders, A., Kreiter, A., Marto, T., Oross, K. & Raczky, P. Innovation or inheritance? Assessing the social mechanisms underlying ceramic technological change in early Neolithic pottery assemblages in Central Europe. In *Detecting and Explaining Technological Innovation in Prehistory* (eds. Spataro, M. & Furholt, M.) 49–71 (Sidestone Press, 2020).

[41] Roux, V. Wheel fashioning techniques: Relative efficiency, technological know-how and symbolic expression. In *La roue et le tour: L'origine de leur adoption en Méditerranée* (eds. Amouretti, S. & Brun, J.-P.) 153–164 (éditions Boccard, 2009).

[42] Motuzaite-Matuzeviciute, G., Lillie, M. & Telizhenko, S. AMS radiocarbon dating from the Neolithic of Eastern Ukraine casts doubts on existing chronologies. *Radiocarbon* **57**(4), 657–664. 10.2458/azu_rc.57.18438 (2015).

[43] Grapes, R. *Pyrometamorphism* (Springer, 2006).

[44] Kertész, R. Mesolithic hunter-gatherers in the Northwestern part of the great Hungarian plain. *Praehistoria* **3**, 281–304 (2002).

[45] Kaczanowska, M. & Kozłowski, J. The origin and spread of the western linear pottery culture: Between forager and food producing lifeways in Central Europe. *Archaeol. Ért.* **139**, 293–318 (2014).

[46] Kaminská, L. Paleolit a mezolit, Archeologický Ústav Slovenskej Akadémie Vied, Nitra (2014).

[47] Shennan, S. *The First Farmers of Europe: An Evolutionary Perspective* (Cambridge University Press, 2018).

[48] Duffy, P. R., Marton, T. & Borić, D. Locating mesolithic hunter-gatherer camps in the Carpathian Basin. *J. Archaeol. Method Theory* **30**(2), 636–677. 10.1007/s10816-022-09570-w (2023).

[49] Evershed, R. P. *et al.* Earliest date for milk use in the Near East and southeastern Europe linked to cattle herding. *Nature* **455**(7212), 528–531. 10.1038/nature07180 (2008). 18690215 10.1038/nature07180

[50] Nieuwenhuyse, O. P., Roffet-Salque, M., Evershed, R. P., Akkermans, P. M. M. G. & Russell, A. Tracing pottery use and the emergence of secondary product exploitation through lipid residue analysis at Late Neolithic Tell Sabi Abyad (Syria). *J. Archaeol. Sci.* **64**, 54–66. 10.1016/j.jas.2015.10.002 (2015).

[51] Debono Spiteri, C., Gillis, R. E., Roffet-Salque, M. & Evershed, R. P. Regional asynchronicity in dairy production and processing in early farming communities of the northern Mediterranean. *Proc. Natl. Acad. Sci.* **113**(48), 13594–13599. 10.1073/pnas.1607810113 (2016). 27849595 10.1073/pnas.1607810113 PMC5137723

[52] Bondetti, M. *et al.* Neolithic farmers or Neolithic foragers? Organic residue analysis of early pottery from Rakushechny Yar on the Lower Don (Russia). *Archaeol. Anthropol. Sci.* **13**(8), 141. 10.1007/s12520-021-01412-2 (2021). 34777611 10.1007/s12520-021-01412-2 PMC8550616

[53] Nordqvist, K. & Kriiska, A. Towards Neolithisation. The Mesolithic-Neolithic transition in the central area of the eastern part of the Baltic Sea. In *The Dąbki site in Pomerania and the neolithisation of the North European Lowlands (c. 5000–3000 calBC)* (eds. Kabaciński, J., Hartz, S., Raemaekers, D. C. M. & Terberger, T.). Rahden/Westf: Leidorf. (Archäologie und Geschichte im Ostseeraum) 537–556 (2015).

[54] Oras, E. *et al.* The adoption of pottery by north-east European hunter-gatherers: Evidence from lipid residue analysis. *J. Archaeol. Sci.* **78**, 112–119. 10.1016/j.jas.2016.11.010 (2017).

[55] Clavel, B. & Arbogast, R. M. Fish exploitation from early Neolithic sites in northern France: the first data. In *The Role of Fish in Ancient Time: Proceedings of the 13th Meeting of the ICAZ Fish Remains Working Group in October 4–9, Basel/August, 2005* (ed. Hüster-Plogmann, H.) 85–89 (Leidorf, 2007).

[56] Lightfoot, E., Boneva, B., Miracle, P. T., Šlaus, M. & O’Connell, T. C. Exploring the Mesolithic and Neolithic transition in Croatia through isotopic investigations. *Antiquity* **85**(327), 73–86. 10.1017/S0003598X00067442 (2011).

[57] Copley, M. S., Berstan, R., Straker, V., Payne, S. & Evershed, R. P. Dairying in antiquity. II. Evidence from absorbed lipid residues dating to the British Bronze Age. *J. Archaeol. Sci.* **32**(4), 505–521. 10.1016/j.jas.2004.08.006 (2005).

[58] Craig, O. E. *et al.* Did the first farmers of central and eastern Europe produce dairy foods?. *Antiquity* **79**(306), 882–894. 10.1017/S0003598X00115017 (2005).

[59] Dudd, S. N. & Evershed, R. P. Direct demonstration of milk as an element of archaeological economies. *Science* **282**(5393), 1478–1481. 10.1126/science.282.5393.1478 (1998). 9822376 10.1126/science.282.5393.1478

[60] Evans, M. *et al.* Detection of dairy products from multiple taxa in Late Neolithic pottery from Poland: An integrated biomolecular approach. *R. Soc. Open Sci.* **10**, 230124. 10.1098/rsos.230124 (2023). 36938542 10.1098/rsos.230124 PMC10014250

[61] Lucquin, A. *et al.* Ancient lipids document continuity in the use of early hunter-gatherer pottery through 9,000 years of Japanese prehistory. *Proc. Natl. Acad. Sci. USA* **113**(15), 3991–3996. 10.1073/pnas.1522908113 (2016). 27001829 10.1073/pnas.1522908113 PMC4839459

[62] Danilenko, V. N. *Neolit Ukrainy: Glavy drevney istorii Yugo-Vostochnoy Evropy [The Neolithic of Ukraine: The chapters of ancient history of the South-eastern Europe]* (Naukova dumka, 1969) (in Russian)

[63] Kiosak, D. *et al.* The last hunter-gatherers and early farmers of the middle southern Buh river valley (Central Ukraine) in VIII–V MILL. BC. *Radiocarbon* **63**(1), 121–137. 10.1017/RDC.2020.120 (2021).

[64] Manen, C. & Mazurié de Keroualin, K. Les concepts “La Hoguette “et” Limburg”: Un bilan des données. In *ConstellaSion. Hommage á Alain Gallay* (eds. Besse, M., Stahl Gretsch, L.-I. & Curdy, P.) 115–145 (Cahiers d‘ Archéologie Romande 95, 2003).

