# Author Correction: Technology and provenience of the oldest pottery in the northern Pannonian Basin indicates its affiliation to hunter-gatherers

**DOI:** 10.1038/s41598-024-75402-4

**Published:** 2024-10-24

**Authors:** Jan Petřík, Karel Slavíček, Katarína Adameková, Victory A. J. Jaques, Martin Košťál, Peter Tóth, Libor Petr, Dalibor Všianský, Tomas Zikmund, Jozef Kaiser, Jozef Bátora, Penny Bickle

**Affiliations:** 1 Department of Geological Sciences, Faculty of Science, Masaryk University, Kotlářská 2, 611 37 Brno, Czechia; 2 Institute of Archaeology of the Czech Academy of Sciences, Brno, Čechyňská 363/19, 602 00 Brno, Czechia; 3 CEITEC ‑ Central European Institute of Technology, Brno University of Technology, Purkyňova 123, 61200 Brno, Czechia; 4 Department of Archaeology and Museology, Faculty of Arts, Masaryk University, Arna Nováka1, 602 00 Brno, Czechia; 5 Department of Botany and Zoology, Faculty of Science, Masaryk University, Kotlářská2, 611 37 Brno, Czechia; 6 Institute of Archaeology, Slovak Academy of Sciences, Akademická 2, 949 21 Nitra, Slovakia; 7 Department of Archaeology, University of York, The King’s Manor, York, YO1 7EP UK

Correction to: *Scientific Reports *10.1038/s41598-024-69208-7, published online 20 August 2024

The original version of this Article contained an error in the Abstract.

“Our findings indicate a non-local provenance, low temperature firing, *Festugc* sp. grass temper and unique rectangular or cylindrical vessel shapes which align with Eastern European hunter-gatherer practices.”

now reads:

“Our findings indicate a non-local provenance, low temperature firing, *Festuca* sp. grass temper and unique rectangular or cylindrical vessel shapes which align with Eastern European hunter-gatherer practices.”

In addition, in the Discussion section,

“Their tempering practices differ regionally; pottery in Pomerania (Poland) included coarse mineral temper of crushed granite^22^, in northern Poland with sand and plants^14^, in the Baltic it was quartzite, sandstone, mineral inclusions, flint, mollusks, less frequently grog (crushed pottery), vegetal temper^14,23^ and shells^25^, mollusks were added to pottery in eastern Ukraine^42^, shells, organic temper or grog in Eastern Europe^21^, bone, shell, minerals, and organics in western Europe’s “so-called hunter-gatherer” potteries from western Europe^17^, or grass in Far East^9^.”

now reads:

“Their tempering practices differ regionally; pottery in Pomerania (Poland) included coarse mineral temper of crushed granite^22^, in northern Poland with sand and plants^14^, in the Baltic it was quartzite, sandstone, mineral inclusions, flint, mollusks, less frequently grog (crushed pottery), vegetal temper^14,23^ and shells^25^, mollusks were added to pottery in eastern Ukraine^42^, shells, organic temper or grog in Eastern Europe^21^, bone, shell, minerals, and organics in “so-called hunter-gatherer” potteries from western Europe^17^, or grass in Far East^9^.”

The original Article has been corrected.

