# Supplementary material for: Technology and provenience of the oldest pottery in the northern Pannonian Basin indicates its affiliation to hunter-gatherers
Source: Sci Rep. 2024 Aug 20;14:19226. doi: 10.1038/s41598-024-69208-7 (PMC11333753; doi:10.1038/s41598-024-69208-7)
Supplement: Supplementary file 1 — Supplementary Information. [file 41598_2024_69208_MOESM1_ESM.docx]

**Methods**

For the purpose of this study, six fragments were thin sectioned, microscopically observed, described and analysed by microtomography, scanning electron microscope (SEM) as well as 3D modelling and subjected to the determination of total organic carbon (TOC) amount. One of the samples (K8) was examined by the means of X-ray powder diffraction (XRD) to identify its bulk phase composition and differential scanning calorimetry and thermogravimetry (DSC/TG) to quantify the remaining organic matter. Moreover, we designed a firing experiment in order to simulate the firing technology and more closely determine the firing temperature. Thin sections of 30 μm thickness were analysed micro-petrographically. The sections were studied under a polarizing microscope Olympus BX 51 according to Whitbread (summarized in Hunt 2017) and Quinn (2013). Inclusion and void abundance were expressed as semi quantities according to Sauer (2005) and Fitzpatrick (1984). Organic content was estimated by the amount of organic shaped pores. Identification of organic inclusions was carried out using anatomical description by G. Martínez-Sagarra (2017). SEM images were carried out by JEOL 6490 LV instrument with ED spectrometer LN2-free. XRD analysis was conducted using a Bruker D8 Advance diffractometer at the conventional reflection geometry. Ni-filtered copper radiation (λKα = 0.15418 nm) was used. The data was processed with the help of the Bruker AXS Diffrac plus EVA 2 and Topas 3 software. The quantitative phase analysis was done by the Rietveld method. TOC was determined as the difference between TC and IC (EN 1484) using TOC-V(CPH) with the module TNM-1 and was used to quantify the amount of charred organic matter in samples. Thermal analysis (DSC/TG) was carried out using a Setaram Setsys Evolution 1750 instrument. The experiment was conducted in a dynamic air atmosphere with a constant heating rate 10 °C/min in the temperature range 40–1000 °C. The acquired data was processed using the Setaram Processing software. X-ray computed micro-tomography (μCT) was used to visualise the organic matter and related porosity. The scanning was performed on the laboratory GE Phoenix v|tome|x L 240 system. The volume was reconstructed, analysed and segmented with Datos|x and VG Studio MAX softwares. We separated the ceramic paste from air and organic matter using the automatic surface determination. We segmented the organic matter from air with a? VG Studio porosity module and refined the segmentation using erosion/dilation filtering (+/- 1 pixel) to clean the data from the smaller particles related to noise. A Nikon D750 camera mounted on a stand and a 60-mm lens were used to capture photos of the sherd under various angles. A virtual copy of the fragment was created using the Structure from Motion method (De Reu et al. 2014 method) and mounted on a stand. Afterwards we carried out the reconstruction of the shape based on reviews of known pottery forms from contemporary Early Neolithic and Late Mesolithic pottery from the Southeast and East European and Asian regions (e.g. Zhushchikhovskaya 2009; Mazurkievich & Dolbunova 2015; Jakucs et al. 2018; Andreev & Vybornov 2021). The software processing took place in the Agisoft Metashape software and the Blender software based on the drawing documentation (cf. Poigt et al. 2021) and then virtually deformed in this software so that the 3D scan could be spatially settled. The experimental firing took place under field conditions, employing a bonfire firing (Orton et al. 1993) designed on the basis of previous experimental firings of R. Thér (2013; 2018). Pottery interspersed with wood (spruce and pine chips), were used to replicate the appearance of discovered pottery. These experimental vessels were made from loess clay, heavily tempered with grass. The loess clay was gathered at Kalinčiakovo village in the close vicinity of Santovka village (N48.1997917, E18.6601175). The grass was cut shorter and mixed into the clay white it was still fresh. The grass temper ratio to clay was 1:2 in volume. The prepared paste was stored for three months. Rather than being formed from coils, they were shaped from whole pieces of clay into a bowl-like form. When the firewood was ignited, it was allowed to burn naturally, without adding more fuel. An infrared thermometer Testo 835-T2 was used to measure the surface temperature of visible vessels. Three samples of pots placed in the centermost section with the supposed lowest availability of oxygen during the experimental firing were used for comparison of organic matter preservation using thin section analysis.

**Methods**

For the purpose of this study, six fragments were thin sectioned, microscopically observed, described and analysed by microtomography, scanning electron microscope (SEM) as well as 3D modelling and subjected to the determination of total organic carbon (TOC) amount. One of the samples (K8) was examined by the means of X-ray powder diffraction (XRD) to identify its bulk phase composition and differential scanning calorimetry and thermogravimetry (DSC/TG) to quantify the remaining organic matter. Moreover, we designed a firing experiment in order to simulate the firing technology and more closely determine the firing temperature. Thin sections of 30 μm thickness were analysed micro-petrographically. The sections were studied under a polarizing microscope Olympus BX 51 according to Whitbread [1] (summarized in Hunt [2]) and Quinn [3]. Inclusion and void abundance were expressed as semi quantities according to Sauer [4] and Fitzpatrick [5]. Organic content was estimated by the amount of organic shaped pores. Identification of organic inclusions was carried out using anatomical description by Martínez-Sagarra [6]. SEM images were carried out by JEOL 6490 LV instrument with ED spectrometer LN2-free. XRD analysis was conducted using a Bruker D8 Advance diffractometer at the conventional reflection geometry. Ni-filtered copper radiation (λKα = 0.15418 nm) was used. The data was processed with the help of the Bruker AXS Diffrac plus EVA 2 and Topas 3 software. The quantitative phase analysis was done by the Rietveld method. TOC was determined as the difference between TC and IC (EN 1484) using TOC-V(CPH) with the module TNM-1 and was used to quantify the amount of charred organic matter in samples. Thermal analysis (DSC/TG) was carried out using a Setaram Setsys Evolution 1750 instrument. The experiment was conducted in a dynamic air atmosphere with a constant heating rate 10 °C/min in the temperature range 40–1000 °C. The acquired data was processed using the Setaram Processing software. X-ray computed micro-tomography (μCT) was used to visualise the organic matter and related porosity. The scanning was performed on the laboratory GE Phoenix v|tome|x L 240 system. The volume was reconstructed, analysed and segmented with Datos|x and VG Studio MAX softwares. We separated the ceramic paste from air and organic matter using the automatic surface determination. We segmented the organic matter from air with a VG Studio porosity module and refined the segmentation using erosion/dilation filtering (+/- 1 pixel) to clean the data from the smaller particles related to noise. A Nikon D750 camera mounted on a stand and a 60-mm lens were used to capture photos of the sherd under various angles. A virtual copy of the fragment was created using the Structure from Motion method (De Reu et al. [7] method) and mounted on a stand. Afterwards we carried out the reconstruction of the shape based on reviews of known pottery forms from contemporary Early Neolithic and Late Mesolithic pottery from the Southeast and East European and Asian regions (e.g. Zhushchikhovskaya [8]; Mazurkevich & Dolbunova [9]; Jakucs et al. [10]; Andreev & Vybornov [11]). The software processing took place in the Agisoft Metashape software and the Blender software based on the drawing documentation (cf. Poigt et al. [12]) and then virtually deformed in this software so that the 3D scan could be spatially settled. The experimental firing took place under field conditions, employing a bonfire firing (Orton et al. [13]) designed on the basis of previous experimental firings of Thér [14, 15]. Pottery interspersed with wood (spruce and pine chips), were used to replicate the appearance of discovered pottery. These experimental vessels were made from loess clay, heavily tempered with grass. The loess clay was gathered at Kalinčiakovo village in the close vicinity of Santovka village (N48.1997917, E18.6601175). The grass was cut shorter and mixed into the clay white it was still fresh. The grass temper ratio to clay was 1:2 in volume. The prepared paste was stored for three months. Rather than being formed from coils, they were shaped from whole pieces of clay into a bowl-like form. When the firewood was ignited, it was allowed to burn naturally, without adding more fuel. An infrared thermometer Testo 835-T2 was used to measure the surface temperature of visible vessels. Three samples of pots placed in the centermost section with the supposed lowest availability of oxygen during the experimental firing were used for comparison of organic matter preservation using thin section analysis.

**References**

1. Whitbread, I. K. (1986). The characterization of argillaceous inclusions in ceramic thin sections. *Archaeometry, 28*(1), 79-88. https://doi.org/10.1111/j.1475-4754.1986.tb00371.x
2. Hunt, A. M. W. (2017). *The Oxford Handbook of Archaeological Ceramic Analysis*. Oxford University Press.
3. Quinn, P. S. (2013). *Ceramic Petrography: The Interpretation of Archaeological Pottery & Related Artefacts in Thin Section*. Archaeopress.
4. Sauer, R. (2005). Semi-quantitative microscopic analysis. In F. Krinzinger (Ed.), *Spätantike und mittelalterliche Keramik aus Ephesos* (pp. 51-57). Österreichisches Archäologisches Institut.
5. Fitzpatrick, E. A. (1984). *Micromorphology of Soils*. Chapman and Hall.
6. Martínez-Sagarra, G. (2017). Study of the leaf anatomy in cross section in the Iberian species of Festuca L. (Poaceae) and its systematic significance. *PhytoKeys, 83*, 43-74. <https://doi.org/10.3897/phytokeys.83.13746>
7. De Reu, J., De Smedt, P., Herremans, D., Van Meirvenne, M., Laloo, P., & De Clercq, W. (2014). On introducing an image-based 3D reconstruction method in archaeological excavation practice. *Journal of Archaeological Science, 41*, 251-262. https://doi.org/10.1016/j.jas.2013.08.020
8. Zhushchikhovskaya, I. S. (2009). Pottery Making in Prehistoric Cultures of the Russian Far East. In P. Jordan & M. Zvelebil (Eds.), *Ceramics Before Farming: The Dispersal of Pottery Among Prehistoric Eurasian Hunter-Gatherers* (pp. 121-148). Left Coast Press.
9. Mazurkevich, A., & Dolbunova, E. (2015). The oldest pottery in hunter-gatherer communities and models of Neolithisation of Eastern Europe. *Documenta Praehistorica, 42*, 13-66. https://doi.org/10.4312/dp.42.1
10. Jakucs, J., Oross, K., Bánffy, E., Voicsek, V., Dunbar, E., Reimer, P., ... & Whittle, A. (2018). Rows with the neighbours: The short lives of longhouses at the Neolithic site of Versend-Gilencsa. *Antiquity, 92*(361), 91-117. https://doi.org/10.15184/aqy.2017.218
11. Andreev, K. M., & Vybornov, A. A. (2021). Ceramic traditions in the forest-steppe zone of Eastern Europe. *Open Archaeology, 7*(1), 705-717. https://doi.org/10.1515/opar-2020-0195
12. Poigt, T., Comte, F., & Adam, L. (2021). How accurate was Bronze Age weighing in Western Europe? *Journal of Archaeological Science: Reports, 40*, 103221. https://doi.org/10.1016/j.jasrep.2021.103221
13. Orton, C., Tyers, P., & Vince, A. (1993). *Pottery in Archaeology*. Cambridge University Press.
14. Thér, R. (2013). Experimental reconstruction of pottery firing techniques. In S. Scarcella (Ed.), *Archaeological Ceramics: A Review of Current Research* (pp. 128-142). Archaeopress.
15. Thér, R., Kallistová, A., & Gregor, M. (2018). Experimental firing of prehistoric pottery under field conditions. *Journal of Archaeological Science: Reports, 17*, 669-679. https://doi.org/10.1016/j.jasrep.2017.12.038
